# Abnormal Pulmonary Artery Stiffness in Pulmonary Arterial Hypertension: *In Vivo* Study with Intravascular Ultrasound

**DOI:** 10.1371/journal.pone.0033331

**Published:** 2012-03-30

**Authors:** Edmund M. T. Lau, Nithin Iyer, Rahn Ilsar, Brian P. Bailey, Mark R. Adams, David S. Celermajer

**Affiliations:** 1 Department of Cardiology, Royal Prince Alfred Hospital, Camperdown, Australia; 2 Department of Respiratory and Sleep Medicine, Royal Prince Alfred Hospital, Camperdown, Australia; 3 Sydney Medical School, University of Sydney, Camperdown, Australia; University of Giessen Lung Center, Germany

## Abstract

**Background:**

There is increasing recognition that pulmonary artery stiffness is an important determinant of right ventricular (RV) afterload in pulmonary arterial hypertension (PAH). We used intravascular ultrasound (IVUS) to evaluate the mechanical properties of the elastic pulmonary arteries (PA) in subjects with PAH, and assessed the effects of PAH-specific therapy on indices of arterial stiffness.

**Method:**

Using IVUS and simultaneous right heart catheterisation, 20 pulmonary segments in 8 PAH subjects and 12 pulmonary segments in 8 controls were studied to determine their compliance, distensibility, elastic modulus and stiffness index β. PAH subjects underwent repeat IVUS examinations after 6-months of bosentan therapy.

**Results:**

At baseline, PAH subjects demonstrated greater stiffness in all measured indices compared to controls: compliance (1.50±0.11×10^–2^ mm^2/^mmHg vs 4.49±0.43×10^–2^ mm^2/^mmHg, p<0.0001), distensibility (0.32±0.03%/mmHg vs 1.18±0.13%/mmHg, p<0.0001), elastic modulus (720±64 mmHg vs 198±19 mmHg, p<0.0001), and stiffness index β (15.0±1.4 vs 11.0±0.7, p = 0.046). Strong inverse exponential associations existed between mean pulmonary artery pressure and compliance (r^2^ = 0.82, p<0.0001), and also between mean PAP and distensibility (r^2^ = 0.79, p = 0.002). Bosentan therapy, for 6-months, was not associated with any significant changes in all indices of PA stiffness.

**Conclusion:**

Increased stiffness occurs in the proximal elastic PA in patients with PAH and contributes to the pathogenesis RV failure. Bosentan therapy may not be effective at improving PA stiffness.

## Introduction

Pulmonary arterial hypertension (PAH) is a group of conditions characterised by similar pathophysiological changes in the pulmonary vascular bed, resulting in pre-capillary pulmonary hypertension, increased right ventricular (RV) afterload and ultimately RV failure.[1] The classical arteriopathy described in PAH involves the distal muscular arteries and arterioles, which are generally regarded as resistance vessels. Endothelial dysfunction, imbalance between vasoconstrictor and vasodilator substances, and smooth muscle proliferation lead to vessel remodeling, loss of vessel cross sectional area, and a progressive rise in pulmonary vascular resistance (PVR).[2]

In clinical practice, RV afterload is often described in terms of PVR but this is only a measure of steady pulmonary pressure-flow relationship and neglects the pulsatile nature of blood flow in any circulation.[3] Thus, assessment of arterial compliance (or its conceptual inverse of arterial stiffness) enables the pulsatile component of RV afterload to be taken into account.[4] In fact, global arterial compliance of the pulmonary circulation and local stiffness of the proximal pulmonary artery have both been shown to be predictors of mortality in PAH from recent studies.[5], [6], [7]

The proximal elastic pulmonary arteries (PA) function as conduit vessels to buffer the pulsatile arterial load and distribute blood flow to the peripheral circulation. Post-mortem histological studies have demonstrated morphological changes in the elastic PA in subjects with PAH.[8] Furthermore, recent animal and human studies using frequency domain impedance analysis have enhanced our understanding of the important contribution of proximal PA stiffness to the pathogenesis of RV failure. [9], [10], [11], [12]

Intravascular ultrasound (IVUS) allows real-time imaging of the morphology and function of the pulmonary vasculature when performed with simultaneous hemodynamic monitoring. It can provide direct assessment of local changes in arterial stiffness as opposed to global changes affecting the entire circulation. To date, only few studies have characterised the mechanical properties of the proximal elastic PA in patients with PAH using the IVUS technique. [8], [13], [14], [15]

Current PAH-specific therapies target the dysfunctional pathways implicated in disease pathogenesis and have predominantly vasodilator effects on the pulmonary vasculature. Bosentan, a dual endothelin receptor antagonist, has been shown to reverse arterial remodeling in animal models of PAH, but its *in vivo* effects on proximal PA stiffness and remodeling have not been studied in humans. Therefore, we sought to use IVUS to determine the local elastic properties of the proximal PA in patients with PAH, and evaluate the effects of 6-months therapy with bosentan on arterial stiffness.

## Methods

### Ethics Statement

The research was conducted according to the principles expressed in the Declaration of Helsinki. The study was approved by the local ethics committee and all subjects provided informed consent.

### Subjects

The study subjects consisted of a healthy control group (n = 8) and a PAH group (n = 8). PAH subjects were assessed as part of a 6-month study that investigated the effect of bosentan therapy on pulmonary endothelial function,[16] who underwent additional IVUS imaging. Inclusion criteria for PAH subjects were diagnosis with idiopathic or connective tissue disease related PAH, no prior PAH-specific therapy, and NYHA Function Class III or IV symptoms. All PAH subjects were prescribed bosentan, a dual endothelin receptor antagonist, for clinical indications and returned for follow-up IVUS assessment and right heart catheterisation after 6-months of therapy. Bosentan was prescribed at a dosage of 62.5 mg twice daily for the first month, followed by 125 mg twice daily. All subjects complied with this dosage schedule. Age and sex matched control subjects were recruited from the cardiac catheterisation laboratory, and underwent IVUS imaging after documentation of normal pulmonary artery hemodynamics invasively.

### IVUS Imaging of Elastic Pulmonary Arteries

Right heart catheterisation was performed via the right femoral vein approach. Right atrial, right ventricular and pulmonary artery pressures were all measured at end-expiration. With continuous hemodynamic monitoring, IVUS imaging of PA were performed using a 40 MHz catheter (Atlantis SR Pro Imaging Catheter, Boston Scientific, Natick, USA), which had an axial resolution of 200 um. IVUS images were displayed by an image console (HP Sonos 1000, Hewlett-Packard, Massachusetts, USA) at a frame rate of 30/secs.

The IVUS catheter was advanced distally into a selected lobar PA, followed by a pullback manoeuvre at a rate of 0.5 mm/sec. If feasible and anatomically accessible, we attempted to image multiple pulmonary lobes in each subject. Twenty-one pulmonary lobes were imaged in PAH subjects; and 13 pulmonary lobes in control subjects. Image quality was inadequate for analysis in one lobe of a PAH subject and also in one lobe of a control subject. Thus, a total of 20 pulmonary lobes from 8 PAH subjects were included in the final analysis (four subjects had two lower lobes and one upper lobe studied; two had one upper and one lower lobe studied; and two had two lower lobes studied). For the controls, a total of 12 pulmonary lobes from 8 subjects were included (three had two lower lobes studied; three had one lower lobe studied; one had one upper lobe studied; and one had upper and one lower lobe studied).

Selective pulmonary angiography was performed to determine the anatomical position of each imaged PA segment. On follow-up studies, the same PA segments were imaged according to baseline angiography. IVUS images were recorded and digitalised for off-line analysis. The accuracy of IVUS-derived measurements of PA diameter, luminal area and wall thickness has previously been validated against in vitro anatomical measurements.[17], [18], [19]

### IVUS-Derived Measurements of Pulmonary Artery Elastic Properties

IVUS image analysis was performed by two study investigators blinded to the clinical status and hemodynamics of subjects, using a commercially available imaging software (Image J Ver 1.44, NIH, USA). The baseline study was cued to the most distal, least-branching segment of the PA with good image quality (defined as complete circumferential demarcation of the intima and wall to adventitia boundaries). The follow-up IVUS was then cued to a point of equivalent PA diameter. Analyses of IVUS images were averaged over 3 consecutive cardiac cycles. The luminal margin PA were traced at end-systole (defined as T wave onset on ECG) and end-diastole (defined as QRS onset on ECG) to determine the end-systolic luminal area (A_s_) and end-diastolic luminal area (A_d_), respectively. Vessel diameters in end-systole (D_s_) and end diastole (D_d_) were derived from the equation; D = 2×√(A/π).

**Figure 1:**
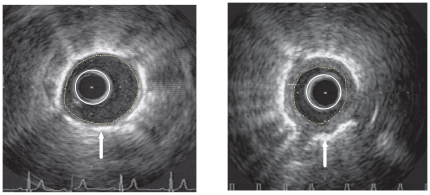
Representative images of an elastic PA with IVUS catheter in situ (white circle). Left image, IVUS image from a control subject with normal wall thickness, as shown by the area bounded by the luminal margin (yellow circle) and the bright elastic lamina (white arrow). Right image, IVUS image from a subject with severe PAH demonstrating a marked increase in wall thickness. Total vessel area was defined by the area circumscribed by the bright elastic lamina; wall area was defined as the total vessel area minus luminal area; and % wall thickness  =  wall area/total vessel area×100.

Using the IVUS-derived measurements and pulmonary hemodynamic acquired at the same procedure (simultaneously), indices of arterial stiffness were used to characterise the elastic properties of PA. Compliance was defined as the absolute area change for a given pressure change (compliance = (A_s_–A_d_)/pulse pressure); distensibility was defined as the relative percentage area change for a given pressure change (distensibility = (A_s_–A_d_)×100/pulse pressure×A_d_ ), elastic modulus as the pressure change required for a theoretical 100% increase in vessel diameter (elastic modulus = pulse pressure×D_d_/[D_s_–D_d_]), and stiffness index β was calculated by the formula (Stiffness Index β = ln(SPAP/DPAP)/[(D_s_–D_d_)/D_d_]).[20] Total vessel area was defined by the area circumscribed by the elastic lamina; wall area was defined as the total vessel area minus luminal area; and % wall thickness  =  wall area/total vessel area×100 (Figure 1).

### Statistical Analysis

Data are expressed as mean±SD unless otherwise stated. Independent sample t-tests were used to compare baseline differences between the control and PAH groups; and paired t-tests were used to compare the effects of bosentan within the PAH group. The association between mean pulmonary artery pressure and stiffness indices were explored using regression analysis, and different curve fitting models were tested and compared with F and R^2^ statistics. A two-sided p value <0.05 was regarded as significant. Analyses were performed on a biostatistics and graphing software (Prism 5, Graphpad, California, USA).

## Results

### Baseline characteristics study populations

The age and gender of PAH subjects and control subjects were well matched (66±4 years vs. 67±4 years; 5 females in each group). In the PAH group (5 subjects with idiopathic PAH, 3 with scleroderma-associated PAH), baseline hemodynamic revealed severe pulmonary hypertension with a mean pulmonary artery pressure (PAP) of 48±3 mmHg, pulmonary capillary wedge pressure (PCWP) of 10±3; and all subjects were in NYHA functional class III. Control group had normal pulmonary hemodynamic with mean PAP of 16±2 mmHg and PCWP of 9±3 mmHg.

### *In Vivo* assessment of pulmonary artery elastic properties with IVUS

At baseline, the mean luminal diameters of PAs were similar in PAH and control subjects (2.5±0.1 mm vs. 2.4±0.1 mmHg, respectively; mean±SEM, p = 0.89). PAH subjects demonstrated increased % wall thickness compared to controls (34±2% vs. 17±2%; mean±SEM, p = 0.001), as previously reported.[16]

**Figure 2:**
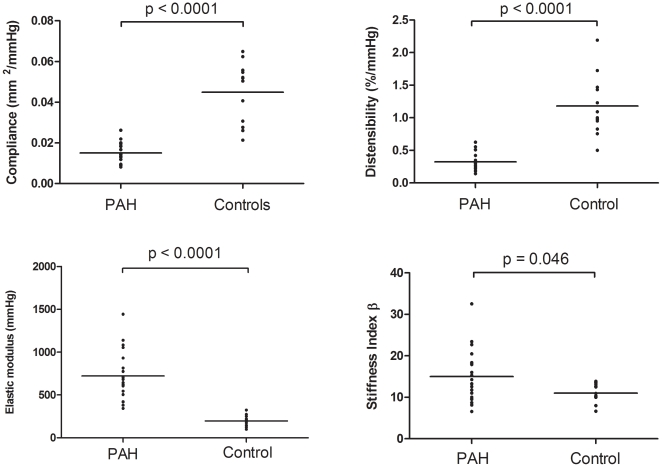
Comparison of PA stiffness indices in control (n = 12) and PAH subjects (n = 20) at baseline. PAH subjects demonstrated significantly greater stiffness in all measure indices; compliance (1.50±0.11×10^–2^ mm^2/^mmHg vs 4.49±0.43×10^–2^ mm^2/^mmHg, p<0.0001), distensibility (0.32±0.03%/mmHg vs 1.18±0.13 %/mmHg, p<0.0001), elastic modulus (720±64 mmHg vs 198±19 mmHg, p<0.001), and stiffness index β (15.0±1.4 vs 11.0±0.7, p = 0.046). Data expressed as mean±SEM.

Compared with controls, PAH subjects demonstrated reduced compliance (1.50±0.11×10^-2^ mm^2/^mmHg vs 4.49±0.43×10^–2^ mm^2/^mmHg; mean±SEM, p<0.0001), reduced distensibility (0.32±0.03%/mmHg vs 1.18±0.13%/mmHg; mean±SEM, p<0.0001), higher elastic modulus (720±64 mmHg vs 198±19 mmHg; mean±SEM, p<0.0001), and higher stiffness index β (15.0±1.4 vs 11.0±0.7; mean±SEM, p = 0.046) (Figure 2).

Of the 20 pulmonary lobes interrogated with IVUS in the PAH group at baseline, only 14 lobes were able to be accessed anatomically and studied at follow-up. If the results were analyzed by consideration of only the 14 lobes that were measured before and after bosentan, we still found a significant difference in all stiffness indices between controls and PAH subjects at baseline.

**Figure 3:**
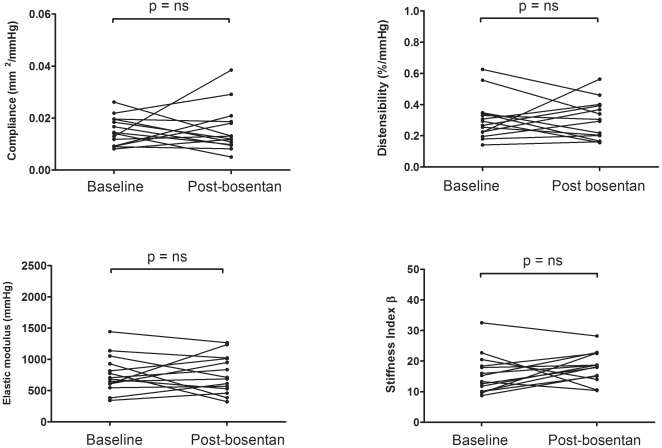
Individual data points of PA stiffness measurements before and after 6-months of therapy with bosentan in PAH subjects (n = 14). Bosentan therapy was associated with no significant changes in PA stiffness indices: compliance (1.51±0.15×10^–2^ mm^2/^mmHg at baseline vs 1.56±0.15×10^–2^ mm^2/^mmHg at 6 months, p = 0.86), distensibility (0.31±0.04%/mmHg at baseline vs 0.30±0.03%/mmHg at 6 months, p = 0.92), elastic modulus (763±80 mmHg at baseline vs 756±81 mmHg at 6 months, p = 0.94), and stiffness index β (15.7±1.7 at baseline vs 18.1±1.3 at 6 months, p = 0.198). Data expressed as mean±SEM.

Following 6-months of bosentan therapy, no significant differences in all indices of PA stiffness were found in the PAH group (Figure 3). Similarly, no significant change in % wall thickness (34±2% vs 36±4%; mean±SEM, p = 0.6) was observed.

**Figure 4:**
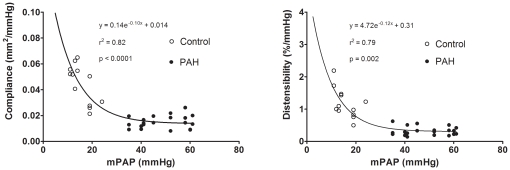
Pooled data points of control subjects (open circle) and PAH subjects (closed circle) showing the strong inverse exponential associations between compliance and mean pulmonary artery pressure (left); and between distensibility and mean pulmonary artery pressure (right). mPAP, mean pulmonary artery pressure.

From the pooled data of all patients, non-linear regression fitted best an inverse exponential relationship between mean PAP and compliance (r^2^ = 0.82, p<0.0001). Similarly, there was a strong inverse exponential relationship between mean PAP and distensibility (r^2^ = 0.79, p = 0.002) (Figure 4).

## Discussion

The present study demonstrates that arterial stiffness is significantly increased in the proximal elastic PA in patients with PAH, and this increase in stiffness occurs in the setting of wall thickening and vessel remodeling. This is also the first human study to evaluate the effects of bosentan on local PA stiffness. We did not observe any significant beneficial effects of bosentan on arterial stiffness following 6-months of therapy.

### Proximal PA involvement in PAH

PAH is regarded traditionally as a disease of the distal small muscular arteries (<500 um in diameter).[21] Progressive obliteration and remodeling of these distal “resistance” vessels eventually lead to elevation in PVR and PAP.[22] The concept of PVR as a determinant of RV afterload and prognosis in PAH has been well studied. However, there is increasing recognition that a decrease in arterial compliance (ie an increase in arterial stiffness) is of equal importance in PAH. Therefore, an understanding of the changes in arterial stiffness that occur in PAH is fundamental to the understanding of progression to RV failure in this condition. Furthermore, it is estimated that about 25% of the total (pulsatile+steady) RV hydraulic load is composed of the pulsatile component, a situation very different to the systemic circulation where it is approximately 10%.[23]

In health, the proximal elastic PA are conduit vessels and function as high capacitance tubes to deliver blood to the peripheral microcirculation. Arterial compliance is most easily and commonly determined by the ratio of the stroke volume to pulse pressure, which describes the compliance of the entire pulmonary circulation. Although useful, this measurement fails to describe regional or local changes in arterial structure and function. Our study, using *in vivo* imaging with IVUS, allowed comprehensive assessment of regional arterial properties and demonstrated that all indices of arterial stiffness are abnormal in the proximal elastic PA in PAH subjects.

The increase in % wall thickness, compared to healthy controls, suggests that these vessels have undergone significant remodeling, and this process of remodeling explains, in part, the increase in arterial stiffness. Stiffness index β has been used as an index of arterial stiffness that is less pressure dependent compared to other commonly used stiffness indices, as the logarithmic transformation adjusts for the non-linear relationship between arterial elasticity and luminal distending pressure.[24], [25] Thus, the increase in stiffness index β observed in PAH subjects supports an intrinsic change in the elastic properties of the PA, as opposed to the sole effect of increased distending pressure *per se*. Since the proximal elastic PA constitute a significant proportion of the total compliance of the pulmonary circulation, our findings indicate that proximal PA stiffening in PAH is an important contributor to the total RV afterload. Our study is in agreement with recent magnetic resonance imaging (MRI) work in humans that has also shown increased proximal PA stiffness in PAH[7], [26], [27], [28], [29], but a key limitation of MRI studies is the lack of simultaneous pulmonary artery pressure measurements to enable detailed analyses arterial stiffness properties.

### Relationship between PA stiffness and PA pressure

The compliance and distensibility data in our subjects demonstrated strong inverse exponential associations with PA pressure. Similar associations were also found by Sanz et al[26] using MRI evaluation of the main PA and hemodynamic data derived from same-day right heart catheterisation. The corollary of this relationship is that at high PAP (such as in subjects with advanced PAH), changes in PAP result in minimal changes in arterial compliance and distensibility. In converse, along the “normal” ranges of PAP (<25 mmHg), small changes in pressure are associated with disproportionately large changes in compliance and distensibility. Thus, as pulmonary vascular disease develops and PAP rises, most of the fall in compliance and distensibility occurs at relatively low PAP. On the other hand. compliance and distensibility reaches a plateau level at around a mean PAP of 40 mmHg. Lankhaar et al[30] have shown that compliance and resistance are inversely related and their product, the resistance-compliance (RC) time constant, remains the same in healthy individuals, PAH subjects, and even in PAH subjects during the course of therapy. [31], [32] Taken together, these observations suggest that early changes in the pulmonary vascular bed are better detected by changes in compliance rather than by changes in PVR or PAP.

The findings of our present study and those of Lankhaar et al may also explain the apparent lack of effect of bosentan on measures of arterial stiffness after 6-months of therapy. Although bosentan may reduce PVR significantly via its vasodilator effect on the distal microvasculature, PAH patients with advanced disease are operating at the flat part of the RC curve, and a significant reduction in compliance would be an unexpected finding even if a large reduction in resistance were to occur. In other words, patients with advanced disease and a higher starting PVR may, with therapy, reduce their PVR with little change in compliance. In contrast, patients with early disease and a lower starting PVR may experience a proportional improvement in both PVR and compliance.

### Study Limitations

The findings of the present study must be interpreted in the context of the following limitations. The main limitation is the small number of subjects in our present study. Although we did not observe any significant differences in PA stiffness following 6 months of bosentan therapy, our study may have lacked sufficient power to detect such a difference. However, a post-hoc analysis indicates that our study was powered to detect a relative change of 40% in PA compliance over time. We were unable to study the same number of pulmonary lobes in all subjects, mainly due to technical difficulties in assessing all arterial territories and the radiation exposure associated with prolonged fluoroscopy times. In this study, the quality of the IVUS image was inadequate for interpretation (>90° of the PA cross section was unclear) in approximately 10% of the PAs studied. Nonetheless, the majority of IVUS images were suitable for analysis and measurements were reproducible. Although IVUS is an invasive imaging modality, right heart catheterisation remains a necessary component of the diagnostic evaluation of subjects with suspected PAH, and IVUS can potentially be performed in the same setting.

### Conclusions

We have demonstrated, using *in vivo* assessment with IVUS, that the proximal elastic PA in patients with PAH undergo remodeling accompanied by a marked increase in arterial stiffness. Thus, pathophysiological changes of the proximal elastic PA play an important contributing role to the overall RV afterload and the development of RV failure in PAH. Despite the known vasodilator effects of bosentan on the distal microvasculature, no change in proximal arterial stiffness was observed following 6-months of therapy. Current PAH-specific therapy may not exert significant beneficial effect on arterial stiffness in subjects with advanced disease, owing to the physiological phenomenon that changes arterial stiffness occur early in PAH. This study further supports the notion that evaluating changes in PA stiffness may have a role in the early detection of pulmonary vascular disease.
